# Apneic uptake of atmospheric O_2_ by deeply hypothermic nestlings of the white-footed mouse (*Peromyscus leucopus*): circulation and lungs

**DOI:** 10.1007/s00360-024-01585-x

**Published:** 2024-10-08

**Authors:** Richard W. Hill, Jacob J. Manteuffel, Bradley A. White

**Affiliations:** 1 Department of Integrative Biology, Michigan State University, East Lansing, MI 48824 USA; 2 Emergency Medicine, Henry Ford Hospital, 2799 West Grand Blvd, Detroit, MI 48202 USA; 3 Center for Youth Development and Intervention, Department of Psychology, The University of Alabama, Tuscaloosa, AL 35487 USA

**Keywords:** Electrocardiography, O_2_ consumption, Lung morphology, Apnea, Ontogeny, Freezing

## Abstract

Nestling white-footed mice (*Peromyscus leucopus*) are born in the earliest days of spring in cold climates. If the nestlings are by accident exposed to ambient temperatures near freezing (0–7 °C) at early ages (2–10 days old), they may experience body temperatures (*T*_b_s) equally low. During such hypothermia, although their heart keeps beating, they become apneic (cease inhaling and exhaling). However, they have an exceptional ability (e.g., compared to *Mus musculus*) to tolerate these conditions for at least several hours, after which they revive if rewarmed by parents. This paper addresses the physiology of the apneic period. We show that apneic, hypothermic nestlings undergo physiologically important exchanges of gases with the atmosphere. These gas exchanges do not occur across the skin. Instead they occur via the trachea and lungs even though the animals are apneic. Most significantly, when hypothermic neonates are in apnea in ordinary air, they take up O_2_ steadily from the atmosphere throughout the apneic period, and the evidence available indicates that this O_2_ uptake is essential for the nestlings’ survival. At *T*_b_s of 2–7 °C, the nestlings’ rate of O_2_ consumption varies quasi-exponentially with *T*_b_ and averages 0.04 mL O_2_ g^− 1^ h^− 1^, closely similar to the rate expressed by adult mammalian hibernators in hibernation at similar *T*_b_s. Morphometric analysis indicates that, at all focal ages, O_2_ transport along the full length of the trachea can take place by diffusion at rates adequate to meet the measured rates of metabolic O_2_ consumption.

**Supplementary Information:**

The online version contains supplementary material available at 10.1007/s00360-024-01585-x.

## Introduction

White-footed mice (*Peromyscus leucopus*) are among the most successful and abundant native rodents in woodlands of the eastern and central United States and in some adjacent parts of Canada (Bedford and Hoekstra 2015). At the northern limits of the species range in places like Michigan and southern Ontario, the species is expanding northward in apparent response to global warming (Myers et al. 2009; Roy-Dufreshe et al. 2013). During each reproductive season in the northern parts of the range, females start giving birth to litters in the earliest days of spring (Long 1973; Millar et al. 1979; Millar and Gyug 1981), when atmospheric air temperatures (*T*_a_s) below 0 °C are common, snow often falls, some soil layers remain frozen, and days of freezing weather often alternate with days of thawing weather (Long 1973). During thawing periods, large quantities of ice-cold melt water, flowing along paths of least resistance, may seep into the forest floor. Although there is almost no empirical knowledge of microclimatic conditions in *P. leucopus* nesting sites, it seems inevitable that early-born litters are sometimes besieged by near-freezing conditions in their nests. Mothers keep litters warm when present (Hill 1972). However, parents are away from litters for many hours per day (Hill 1972; Harland and Millar 1980). During such parental absences, a nestling in its nest may experience a deep body temperature (*T*_b_) near freezing (Hill 1976).

Already in 1976, Hill (1976) documented that *P. leucopus* nestlings aged 2–10 days have a remarkable ability to tolerate and survive near-freezing *T*_b_s in the range of 0–5 °C, a condition we term “near-freezing hypothermia” or “deep hypothermia.” The tolerance of *P. leucopus* nestlings to low *T*_b_s substantially exceeds the tolerance of both feral and inbred (i.e., laboratory) *Mus musculus* nestlings of similar ages and the tolerance of inbred *Rattus norvegicus* nestlings (Hill 2017). If a *P. leucopus* nestling’s *T*_b_ falls to about 7 °C or lower, the youngster becomes comatose (unresponsive to external stimulation), and it enters a state of apnea, which we define to be an absence of active, mechanical breathing (inhalation and exhalation) (Hill 1976). This unresponsive, apneic state can then continue for at least 3–4 h without jeopardizing successful recovery. Moreover, the only requirement for recovery is rewarming (Hill 1976). Thus, if the young animal’s parent returns and rewarms the nestling, the youngster will spontaneously resume breathing (autoresuscitate) and recover normal motor functions. Thereafter, the youngster will complete the rest of its nestling development in the usual way (Hill 2000, 2017). Quite possibly this ability to recover from near-freezing hypothermia is an adaptation to the threat of such hypothermia. The loss of function during hypothermia would be lethal if not reversed, but it is in fact readily reversed because simple rewarming is the only requirement.

The principal purpose of this paper is to address the physiological properties of *P. leucopus* nestlings during the deep-hypothermia apneic period. This period is defined to start when a nestling – subjected to hypothermia – ceases active, mechanical breathing. The period is defined to end when the nestling, during rewarming, spontaneously takes its first breath; this first inhalation is typically a sudden, highly visible, and massive effort, and over ensuing minutes, the neonate invariably and gradually settles into a pattern of ordinary mechanical breathing (Hill 1976). Under the conditions of most of our experiments (e.g., *T*_b_ = 2–3 °C during hypothermia, rewarming by exposure to warmer air, *T*_a_ ≅ 20 °C), the duration of the apneic period was about 3–3.5 h.

The studies we here report had their beginning in a “lucky accident to the prepared mind.” Often, after a rewarming nestling takes its first breath, its heart rate promptly increases. We were doing experiments, entailing electrocardiographic (EKG) recording, to determine the cause of this cardioacceleration. The experiments were based on a manipulation of the gas inhaled in the first breath: ordinary air or nitrogen (N_2_). To arrange for a rewarming nestling to inhale N_2_ in its first breath, we placed the apneic individual in N_2_ about 10 min in advance. After just a few experiments with N_2_, we became convinced that the EKG was being affected by the N_2_ atmosphere (in ways later described) prior to the animal’s taking its first breath. That is, the EKG of the *apneic* nestling was affected if the nestling’s atmosphere was N_2_ instead of air. This was our “lucky accident,” and the rest of this paper documents the many studies we have done to understand the phenomenon and its implications. The studies include further EKG experiments in air and N_2_, based on the premise that if an apneic nestling is exchanging gases with the atmosphere despite its apneic state, the nestling’s myocardial O_2_ partial pressure could be affected by atmospheric composition. Thus EKGs could provide evidence for or against apneic gas exchange.

Two background points are important for understanding the design and interpretation of our studies. First, pacing of the heart rhythm by the sinoatrial node ceases as a nestling enters deep hypothermia (Hill and Manteuffel 2012).^1^ Nonetheless, the heart of the nestling continues to beat in a regular or quasi-regular rhythm because an ectopic pacing site takes over (Hill and Manteuffel 2012). In commenting on heart action during times when these conditions prevail, we refer to the electrocardiographic signature of ventricular contraction as the “ventricular waveform,” rather than “QRS complex,” precisely because the concept of the QRS complex is associated with sinus pacing. An important practical implication of ectopic pacing is that an ectopic pacing site is not as stable as the sinoatrial-node pacing site in its physical location or in the electrocardiographic waveform it initiates (see Hill and Manteuffel 2012; in which pertinent EKG traces are presented). We have had to take this consideration into account throughout our analysis of our EKG data.

The second important background point to make is that as *P. leucopus* nestlings mature between birth and the age of weaning (ca. 21 days; Millar et al. 1979), they begin at about 12 days of age to express a dramatically increasing capacity for thermoregulatory thermogenesis and, thus, ability to defend against hypothermia (Hill 1976). We focus here on nestlings aged 2–10 days because this age range is the period of their lives when they are most likely to experience deep hypothermia.

As Hill et al. (2021) have stressed, the physiology of young, developing animals needs to be an important focus for environmental and comparative physiologists because, demographically speaking, the developing young of a species living in the wild are typically the most abundant age class. In their remarkable demographic analysis of a free-living, wild population of *P. leucopus*, Goundie and Vessey (1986) found that half of all individuals born in the population spent their entire lives between birth and 1–2 weeks of age. They never reached adulthood but instead were never-adults that existed only as nestlings (Hill et al. 2021).

## Materials and methods

### Animals

Parents of the nestlings studied were either wild-caught or ≤ 4 generations removed from wild-caught *Peromyscus leucopus* captured in forests near East Lansing, MI, and Middletown, CT. They were housed as mated pairs in polycarbonate cages with wood-shaving bedding and cotton for nest construction at ca. 22 °C and on a 16 L:8D photoperiod. Food (Purina Laboratory Animal Chow) and water were provided *ad libitum*.

Pregnant females were checked every day to see if they had given birth, meaning that ages of young were accurate to within a day. We considered the day of birth to be age 0. Body weights of the young were measured with a Mettler P1210 balance. In each experiment reported, a deliberate effort was made to maximize the diversity of parentage of the young studied, within the constraint of animal-colony size. After an experiment, young studied were placed in an incubator at *T*_a_ = 32 °C for ≥ 30 min prior to being returned to their parents.

### Statistics

Statistical analyses were carried out in IBM SPSS Statistics (version 29) except where stated otherwise.

### Evidence for apnea

To verify apnea, many hours of visual observations were made of young in deep hypothermia (e.g., in the minutes immediately after experiments, when the young had been removed from apparatuses, and therefore were easily visible, yet were still hypothermic). More that 10 people, who helped carry out these studies or assisted with them, closely observed numerous brightly illuminated hypothermic nestlings visually to see any breathing movements that occurred. In addition, during EKG studies electrical potentials in the EKG leads were recorded at high enough gain for electromyographic potentials generated by the inspiratory muscles to be typically very evident in the EKG record when nestlings were observed to be inhaling and exhaling. To enhance detection of such potentials, an audio monitor (Grass AM5) was attached in parallel to the recorded potentials so that electrical signals were audible; for example, when a nestling took its first breath after an episode of apnea, a loud, rumbling audio signal was invariably heard following the preexisting silence. Electrical and audio signals were closely monitored throughout all EKG experiments to detect if and when breaths were taken.

### Measurement of rate of O_2_ consumption

**Fig. 1:**
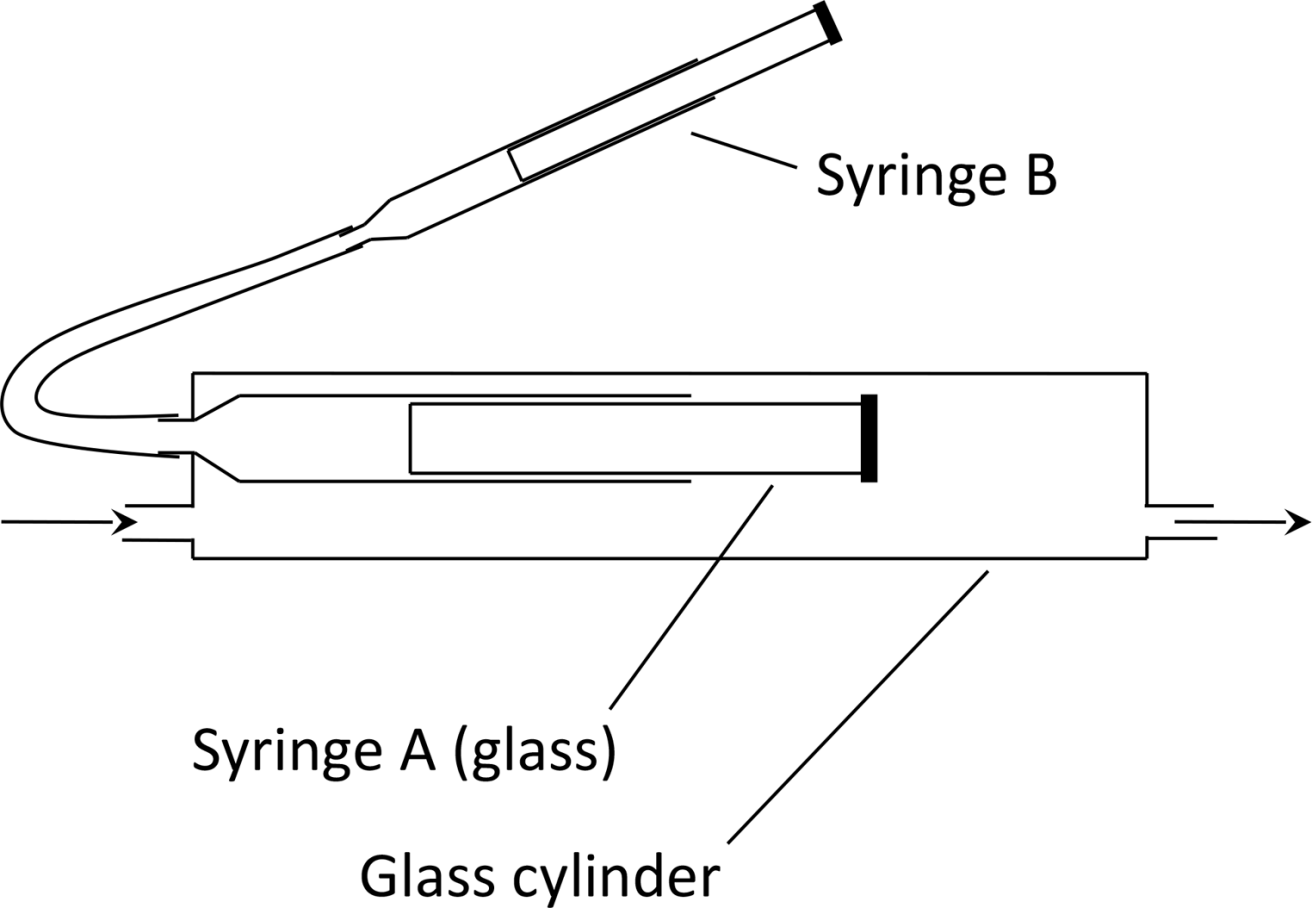
Schematic diagram of pressure-stabilizing device. A non-lubricated glass syringe (**A**) is mounted inside a glass cylinder as shown (we used a 3 mL Becton Dickinson syringe, and our glass cylinder was just slightly greater in diameter than the syringe). As air circulates inside the closed respirometry system, it passes through the glass cylinder, as symbolized by arrows. At the beginning of a period of measurement, the plunger in syringe **A** is maximally inserted in the syringe barrel (i.e., shifted fully to the left). As metabolism of the animals reduces the moles of gas inside the closed system, syringe **B** is used to force the plunger of syringe A to emerge further from the barrel, decreasing the total volume available for the moles of gas to occupy. We monitored the pressure in the gas inside the closed-circuit respirometry system relative to room pressure (barometric pressure) by use of a water manometer, and, using the illustrated device, kept the inside pressure constant for the duration (ca. 1 h) of the measurement period required for definition of nestling O_2_ consumption. Barometric pressure (monitored with a mercury barometer) was required to remain stable for that duration

For high-precision measurements of low rates of O_2_ consumption by hypothermic nestlings, we devised an apparatus modeled on the closed-circuit system of Depocas and Hart (1957), in which air is recirculated without resupply from the outside, meaning that depletion of O_2_ from the air is cumulative, permitting very low rates of O_2_ consumption to be quantified. For measurement, nestlings were placed in an animal chamber consisting of a 10-cm-long length of 3.8-cm-diameter (OD) glass tubing. The components of the complete respirometry system were connected together entirely by lengths of 2.8-mm (OD) glass tubing or 3-mm (OD) stainless-steel tubing except that short (≤ 15 mm) lengths of 2.4-mm (ID) Tygon R-3603 tubing were used as flexible connectors between the lengths of glass or stainless-steel tubing. The following were connected in series: (1) an Applied Electrochemistry S-3 A O_2_ analyzer using a N-22M O_2_ sensor; (2) a Cole-Parmer Masterflex peristaltic pump (including a 21-cm length of compressible tubing, Tygon R-3603) to circulate air within the closed system; (3) the animal chamber; (4) a glass pressure-stabilizing device (Fig. 1, described shortly); (5) a vertical glass reagent tube containing Drierite (for moisture removal) and Ascarite (for CO_2_ removal); and (6) for measuring the rate of gas circulation in the system, a calibrated glass rotameter (Porter tube A-125-7) positioned just upstream of the O_2_ sensor. The pump pulled air through the sensor and pumped it into the animal chamber, where the air flowed the length of the chamber tube before proceeding onward. The animal chamber was located inside a custom-built, precision temperature-regulating cabinet, which controlled *T*_a_ inside the chamber (measured by a calibrated copper-constantan thermocouple connected to a Honeywell class 15 recording potentiometer) to within +/- 0.2 °C.

Because the O_2_ partial pressure measured by the S3A O_2_ analyzer is exquisitely sensitive to total pressure in the gas stream, the gas hydrostatic pressure inside the closed respirometry system was held constant by use of an all-glass pressure-stabilizing device (Fig. 1). During each study, to test for stability of the measurement characteristics of the S3A, an aliquot (1200 mL) of room air was stored in a Brooks Vol-u-meter, and portions of that single aliquot were passed through the O_2_ sensor at the start and end of the measurement period. To ensure absence of leaks in the respirometry system, tests were performed by filling the system with moderately O_2_-depleted air and monitoring the measured O_2_ partial pressure for periods of hours without animals present.

Nestlings aged 3–10 days were studied as sets of littermate siblings. After a litter was removed from its parents for study, individual members of the litter were initially placed in separate containers at the *T*_a_ to be studied (within the range 0–7 °C) for 1.0–1.5 h, during which they became apneic and comatose. Then 2–4 littermates – sufficient for the total body weight of the studied group to be about 7–12 g (to provide a robust O_2_ signal) – were placed in the animal chamber. The individual littermates were spaced out along the length of the chamber, and being comatose, remained spaced out during study. After sealing of the respirometry system, the partial pressure of O_*2*_ in the recirculating air was recorded continuously for 2.0–2.5 h on a Houston 4900 SuperScribe recorder.^2^ For calculation of the nestlings’ rate of O_2_ consumption, the total volume of the air space in the sealed respirometry system had to be known. In separate calibration studies, we measured this volume (158 mL) by bolus injection of N_2_ (Depocas and Hart 1957).^3^

### Studies of O_2_ consumption by apneic nestlings with mouth and nares reversibly occluded

After establishing that apneic nestlings consume O_2_, we hypothesized that the uptake of O_2_ occurred via the trachea and lungs (rather than across the skin). To test this hypothesis we sought to do occlusion experiments, and in a series of preliminary studies, we designed a method to reversibly occlude the mouth and nares of a comatose, hypothermic nestling. A layer (ca. 2 mm thick) of surgical cotton was placed over the mouth and nares, then was saturated with water (but not oozing or dripping wet) and held in position with a sheet of ultra-thin aluminum foil folded over the nestling’s muzzle. This occlusion device could be applied in a few tens of seconds and removed instantly.

We carried out experiments on seven litters of 6- to 10-day-old nestlings at *T*_a_ = 2 °C. Nestlings (2–3 per litter) were placed in the animal chamber for measurement of O_2_ consumption as earlier described. After 2.3 h at the target *T*_a_, the chamber was opened, and working as rapidly as possible, we removed each nestling, applied occluding cotton, and returned all nestlings to the chamber. During the procedure, a gentle stream of cold air (1–5 °C) was directed over the animals to ensure that hypothermia was maintained. The process required ca. 5 min, after which we closed the animal chamber and resumed measurement of O_2_ consumption for 45–50 min, of which the initial 15 min were discounted because of the transitional changes in conditions of measurement. The study was then ended by removing the cotton and transferring the nestlings to *T*_a_ ≅ 20 °C for recovery.

### Lung morphometric studies

To obtain data permitting estimation of rates of diffusion in major pulmonary airways, lung morphometric studies were carried out on nestlings aged 2–22 days. After euthanasia of a nestling in an atmosphere of CO_2_, a longitudinal ventral incision was made in the skin of neck and thorax, and the rib cage, heart, and other tissues were removed as needed to expose the principal pulmonary airways for measurement by digital image processing. Measurements were carried out on the fresh (unfixed) preparation, avoiding potential fixation artifacts. Dimensions of the trachea were measured first. A canula was then inserted in the upper end of the trachea via the glottis, and gentle, minimal air pressure was applied while measurements were made on the other principal airways (see Results). A Javelin Electronics JE7362 CCD camera fitted with a Javelin 884,926 macro zoom lens (12.5–75 mm, f1.8) was used for imaging, and digital image processing was carried out at optimal magnification using BioScan Optimas software (version 4.02) in conjunction with an Imaging Technology PCVision*plus* frame grabber and Sony high resolution video monitor.

The length of the trachea was measured from the anterior margin of the laryngeal skeletal elements to the point of bifurcation of the primary bronchi (specifically, the position of the anterior wall of the right primary bronchus, almost identical to the position of the posterior wall of the left primary bronchus). Because of the importance of tracheal diameter to diffusion calculations, outside tracheal diameter was measured in two ways, recognizing the need to take account of any variation in tracheal diameter along the length of the trachea. First, outside diameter was measured at two places, near the glottis and near the branch point to the primary bronchi, and these were averaged. Second, a long, narrow trapezium was drawn to match the outside borders of the trachea along its full length, and tracheal diameter was calculated by dividing the area of the trapezium by tracheal length. The outside diameter of each bronchus was measured at the middle, although when diameter perceptibly varied along bronchus length, two measures were made near the two ends and averaged. To permit calculation of inside diameters of trachea and bronchi, wall thickness of both elements was measured by focusing to visualize the element in sagittal section and measuring the wall.

### Electrocardiographic (EKG) studies: nestlings during whole-body exposure to air or N_2_

As noted in the Introduction, we had learned from preliminary experiments that atmospheric composition (air or N_2_) can exert cardiac effects in apneic nestlings, meaning that EKG studies can provide insight into apneic gas exchange. Thus we included further EKG studies in the present research.

For preliminary cooling, a nestling was placed alone on a circular (7.5 cm diameter) platform of 3.2-mm-mesh hardware cloth inside a pre-cooled, ventilated glass bell jar (8 cm inside diameter, 8 cm high, with a flat acrylic top) at an air temperature (*T*_a_) of 2–3 °C. The platform was elevated about 4 mm above the base on which the bell jar rested. Two ports in the acrylic top of the bell jar served as inflow and outflow ports for steady air flow through the chamber. To monitor *T*_a_ in the bell jar, a copper-constantan thermocouple, connected to a Honeywell Class 15 recording potentiometer, was positioned in the gas space inside the jar via a third port. The entire set-up was placed in the precision temperature-regulating cabinet for control of *T*_a_. The “start time” for an episode of hypothermia was considered to be the clock time at which *T*_a_ in the bell jar fell to be within the target range of 2–3 °C.

At about 1.3 h after start time, the young animal had become comatose, and the apparatus was briefly removed from the temperature-regulating cabinet to enable attachment of electrodes for EKG recording. Throughout this procedure (lasting 4–5 min), which entailed lifting the bell jar off the hardware-cloth platform, a gentle flow of cold air was maintained over the animal as earlier described. The nestling was positioned with its legs spread, dorsal-side-up on the platform, and Grass Instruments E2 platinum needle electrodes were inserted subcutaneously at four sites: right and left forelegs, left hindleg, and mid-back (used as ground). The bell jar was then again lowered over the platform (electrode leads exiting under the lower lip of the jar). For electromagnetic shielding, the apparatus was placed inside a cubic Faraday cage (28 cm in each dimension) constructed of grounded 1.5-mm-mesh copper screening. The cage was then placed in the temperature-regulating cabinet, resting on foam rubber for vibration isolation. For EKG recording, the leads of the E2 electrodes were connected to a Grass 79 C polygraph with 7P5 preamplifier and 7DAE driver amplifier. Extensive electrical shielding and mechanical stabilization were employed at all steps in signal transmission (e.g., the bundle of electrode wires was wrapped in grounded aluminum foil).

Beginning at about 2.3 h after start time, the hypothermic animal was exposed to two or three gases (air or pure N_2_) in sequential steps: A, B, and sometimes C. In EKG Series I, we studied 14 nestlings, and steps A, B, and C consisted of exposure to air, N_2_, and air, respectively. In EKG Series II, we obtained data on 15 different nestlings studied in two steps, with steps A and B being N_2_ and air, respectively. Each step was planned to last 30 min, but exposures to N_2_ were terminated early if signs of potential imminent cardiac arrest (e.g., pronounced bradycardia or frequent skipped beats) became evident in the EKG. Whether the gas was air or N_2_, the routine flow rate through the bell jar was 680 mL min^− 1^ (measured at room conditions). When one gas was switched to the other (e.g., air switched to N_2_), however, the change was started with a purge at high flow rate (see following section, *Gases and gas purges*). After completion of all steps, the young animal was detached from the EKG electrodes and placed in room air at room temperature (ca. 20 °C) until it spontaneously started to inhale and exhale, prior to being placed in the incubator at *T*_a_ = 32 °C and returned to its parents.

One focus for analysis of results was the transition from air to N_2_. Data for this analysis were obtained for the 14 nestlings in Series I (steps A and B). The second focus for analysis was the transition from N_2_ to air. Data for this analysis were obtained for 29 nestlings: the 14 nestlings in Series I (steps B and C) and the 15 different nestlings in Series II. For all nestlings, their exposure to N_2_ in these experiments was the first time they had ever been exposed to N_2_.

### Gases and gas purges

For experiments with N_2_, we used AGA Specialty Gases NF medical-grade N_2_. All gas flow rates were measured with Brooks or Gilmont rotameters individually calibrated using a Brooks Vol-u-meter.

As earlier noted, a steady rate of gas flow through the animal study chamber (glass bell jar) at 680 mL min^− 1^ was maintained at all times. For switching from air to N_2_, or vice versa, however, the new gas was initially delivered at a higher rate to minimize the time required for the transition. Bell jar volume was ca. 420 mL. At the start of a transition, the new gas was passed through the bell jar at 6 L min^− 1^ for 20 s, sufficient to accomplish 99% conversion to a pure atmosphere of the new gas according to the formulas of Lasiewski et al. (1966). After the purge the routine flow rate was restored.

### EKG studies: nestlings during masked studies in which face and body could be exposed to different gases

**Fig. 2:**
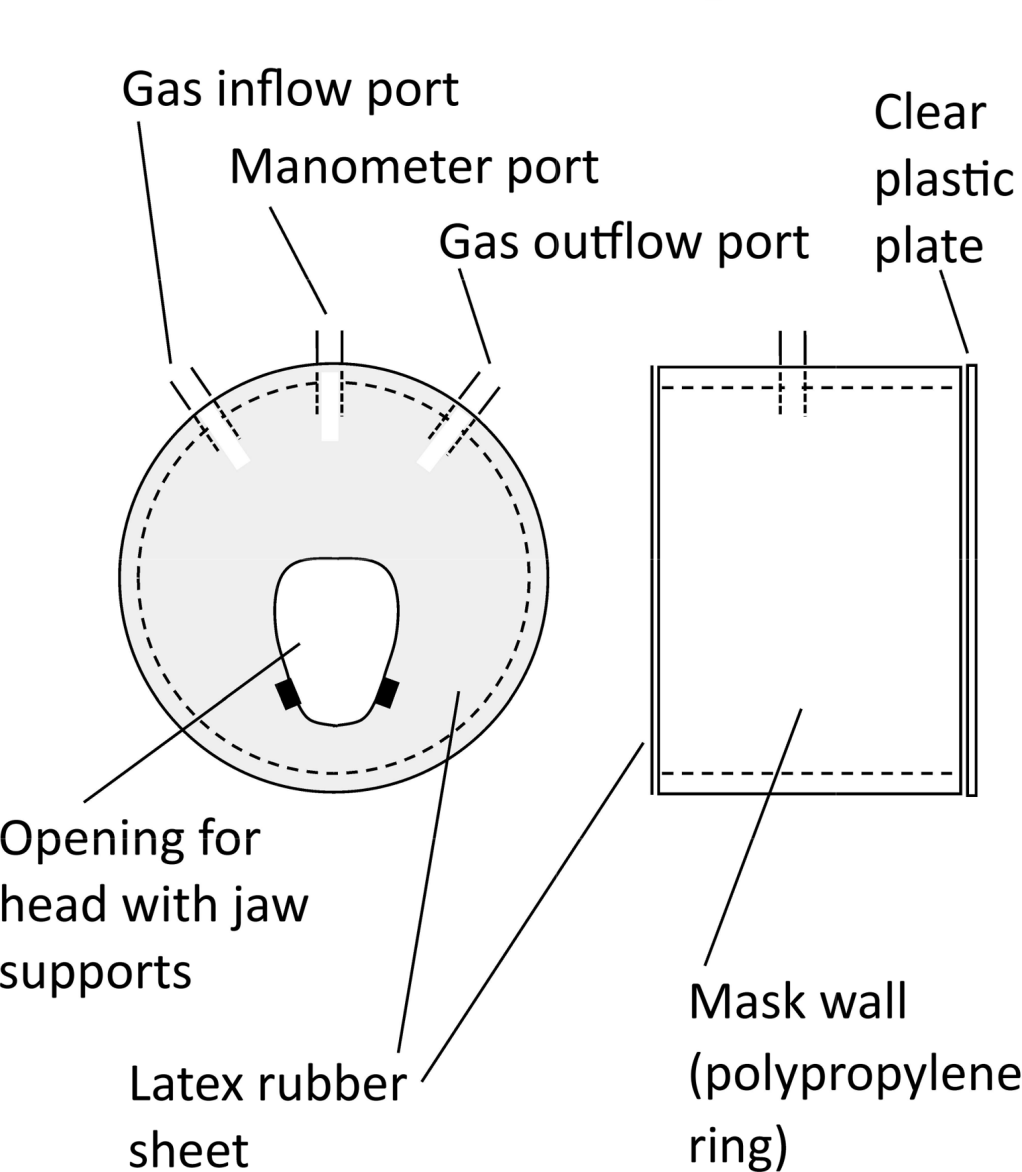
Schematic diagram of mask (not drawn exactly to scale). Mask was cylinder-shaped: At left is view of the cylinder end covered with a thin sheet of latex rubber; at right is side view of the cylinder. The outside diameter of the polypropylene ring was 2.9 cm (2.5 cm ID). Width of the ring was 2.1 cm. The size and exact shape of the opening for the nestling’s head in the latex sheet were designed so that the edges of the opening closely matched the studied nestling’s head. Small blocks of wetsuit foam rubber (4 mm x 7 mm x 2.5 mm thick; shown here as small black rectangles) were attached to the rubber sheet as “jaw supports” to help stabilize the nestling’s head in a vertical position. Ports were made of 5-mm-diameter (OD) glass tubing. The sheet of latex rubber (cut from a surgical glove) was mounted on the polypropylene ring by being folded over the edges and held in place by a tight retaining ring (not shown)

The aim of these studies was to identify the part of the body (face or body posterior to face) where the composition of the atmosphere exerts its effects: that is, the part of the body where gas exchange with the atmosphere occurs in an apneic nestling. In these experiments, we used a mask to enclose the neonate’s face, including mouth and nares. The mask consisted of a 2.1-cm-wide ring of 2.9-cm-diameter (OD) polypropylene tubing sealed on one side with a sheet of clear plastic and on the other with a thin sheet of latex rubber (Fig. 2). A head-opening was cut in the rubber sheet (see Fig. 2) at a size to match the head of the nestling being studied, such that the edges of the opening made contact with the head without exerting constrictive pressure. When a nestling was wearing the mask, its mouth and nares were exposed to the gas flowing through the mask, whereas the rest of its body was exposed to the gas flowing through the glass bell jar, hereafter termed the “body chamber.”

For design of this apparatus, our primary consideration was to avoid differences of gas hydrostatic pressure between the gas spaces in the body chamber and mask because hydrostatic pressure differences, if permitted, could potentially cause ventilation of the lungs or ventilation of other respiratory passages. Thus the opening in the rubber sheet for the nestling’s head was cut to leave a slight gap between the underside of the lower jaw and the lower edge of the opening, permitting free intercommunication between the gas spaces. Small foam rubber jaw supports (see Fig. 2) helped ensure that the head would stay in the upper part of the opening. Because of the design of the mask, gas flowing through the mask could potentially exit in part into the gas space of the body chamber. To minimize any contamination of the body-chamber gas with mask gas, the routine rate of flow through the body chamber during the mask experiments (except during purges) was 1050 mL min^− 1^, which was 15 times the rate of flow through the mask (70 mL min^− 1^). The absence of gas hydrostatic-pressure differences between the gas spaces in body chamber and mask was verified by use of a water manometer, one side connected to the gas space in the mask (see Fig. 2), the other connected to the gas space in the body chamber.

During the early stages of an experiment, the nestling was handled as described in the earlier EKG experiments, except that when the EKG electrodes were attached, the mask was also applied: The nestling’s muzzle was slipped into the opening in the latex sheet of the mask (Fig. 2) up to the level of the eyes, and the position of the mask was stabilized using miniature bungee cords attached to the hardware-cloth platform on which the nestling rested. As soon as the EKG leads and mask were in place, we placed the body chamber (with air flowing through it) over the platform (with mouse resting on top) and started air flow through the mask. We then returned the apparatus to the Faraday cage and the temperature-regulating cabinet (*T*_a_ = 2–3 °C).

The apparatus was designed so that either air or N_2_ could be passed through the mask, and likewise either air or N_2_ could be passed through the body chamber. For switching the type of gas flowing through the body chamber, the purge technique (see *Gases and gas purges*) was used. However, the small volume of the gas space in the mask (ca. 10.3 mL) meant that a purge was unnecessary to achieve a rapid transition in the mask gas. A short length of small-diameter Tygon tubing (3 mm ID), which washed out in 20 s, was used to supply gas to the mask, and at the routine mask flow rate (70 mL min^− 1^), 99% turnover of the gas inside the mask was achieved in 44 s (Lasiewski et al. 1966).

Beginning at 2.3 h after start time, the apneic, hypothermic nestling (3–11 days old) was exposed to a sequence of gas combinations. As before, each step was planned to last 30 min, but steps were terminated early if the EKG suggested potential imminent cardiac arrest (e.g., by development of profound bradycardia). Three principal protocols, which we term EKG Series III, IV, and V, were used. In EKG Series III (22 nestlings studied) the steps were Step A: N_2_ flowing through the body chamber while air flowed through the mask, Step B: air flowing through both body chamber and mask. In EKG Series IV (5 nestlings) the steps were Step A: N_2_ flowing through the mask while air flowed through the body chamber, Step B: air flowing through both mask and body chamber. In EKG Series V (18 nestlings) the steps were Step A: N_2_ flowing through the mask while air flowed through the body chamber, Step B: air flowing through the mask while N_2_ flowed through the body chamber.

Fourteen individuals studied in EKG Series III, a two-step protocol, were subjected to a third step: specifically Step A: N_2_ flowing through the body chamber while air flowed through the mask, Step B: air flowing through both body chamber and mask, and Step C: N_2_ flowing through the mask while air flowed through the body chamber. The concept of this series (EKG Series VI) was to examine the responses of single individuals to both face-only N_2_ and body-only N_2_ within a short span of time, under test conditions that did not substantially suppress heart action prior to either test (by the time we did these experiments we knew that N_2_ in the body chamber had little effect).

### Data extraction from EKG records

Data were read from EKG charts in successive 2-min intervals for the duration of each experiment. The data for each 2-min interval included average heart rate during the interval (number of ventricular waveforms min^− 1^) and average amplitude of the ventricular waveforms (measured in µV as the absolute difference between the most positive and most negative parts of the ventricular waveforms).

As noted in the Introduction, pacing during deep hypothermia was by ectopic sites that could change from time to time. This fact introduced an essential consideration for data analysis, namely that a change in the operative ectopic site could itself cause changes in measured heart rate or amplitude. To avoid such artifacts, we found it to be essential to scan EKG records closely in their entirety, beat by beat, prior to extracting data. If this beat-by-beat analysis revealed a change of ventricular waveform likely to reflect a change in pacing site, data for the affected record (or segment of a record) were not used. A single person carried out this data extraction for all records.

In cases when a nestling was switched from conditions that caused N_2_-mediated cardiac suppression into conditions that permitted recovery, there was sometimes a lag of 2–4 min (rarely 6–8 min) before recovery began. When lags like this occurred, we have included data from the lag period in calculating the extent of cardiac suppression.

## Results

### Animals

Other than nestlings euthanized for the study of morphometrics, studied young were returned to their parents following study. With rare exceptions, they completed their nestling life to the age of 21 days (when parents and young were separated), documenting parental acceptance and continued neonate nursing behavior.

### Evidence for apnea

Based on tens of hours of electrical, audio, and visual monitoring by > 10 observers, we saw no evidence of mechanical ventilation (inhalation and exhalation) in nestlings we classed as apneic.^4^

### Rate of O_2_ consumption by apneic nestlings

**Fig. 3:**
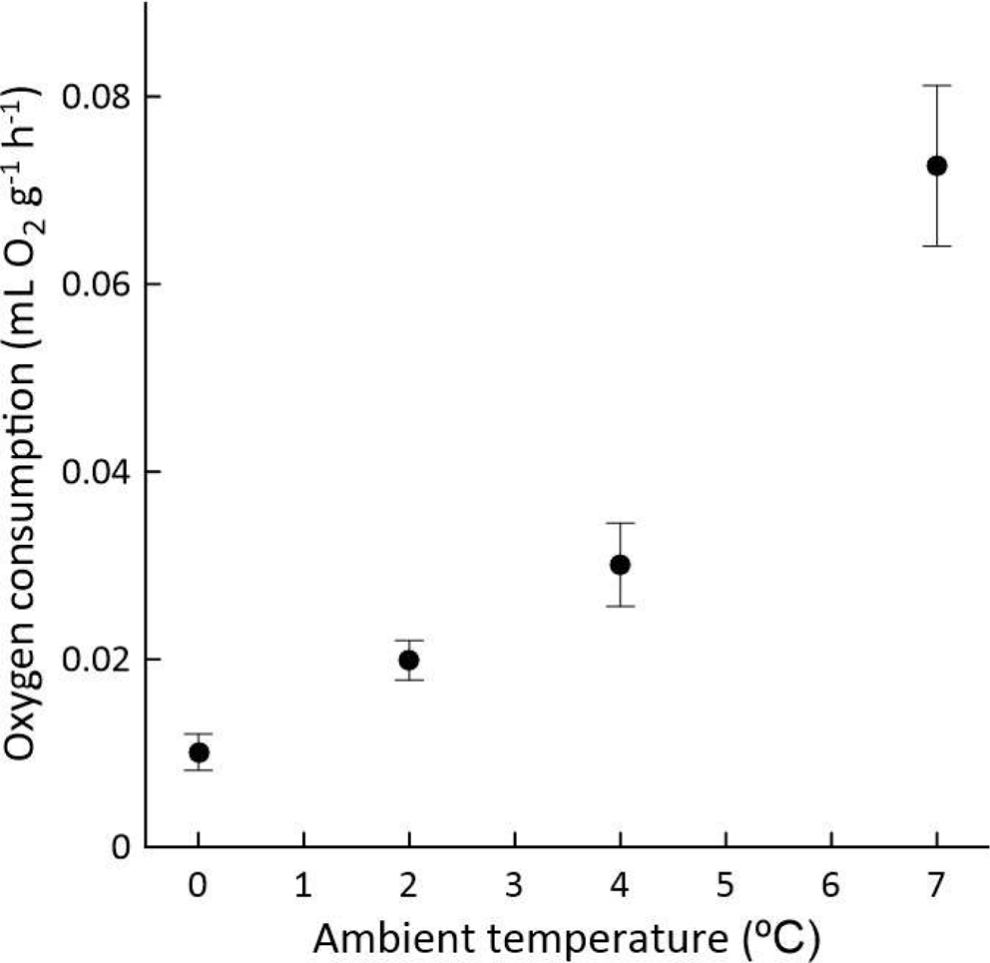
Rate of O_2_ consumption (expressed at STP) by apneic 3- to 10-day-old nestlings as a function of *T*_a_: mean and standard deviation. Although nestlings were studied as sets of litter-mate siblings, the individuals in each set were not in contact with each other. Each litter used in these studies was studied only once, at a single *T*_a_. The number of litters studied was 14, 18, 6, and 6 at *T*_a_ = 0 °C, 2 °C, 4 °C, and 7 °C, respectively. In each study, although data were recorded continuously, the data shown here are for a 1-hour-long period starting 2.3 h after nestlings were placed at the test *T*_a_

All studied nestlings, which were comatose and apneic during the measurement period, consumed O_2_ at a steady, uninterrupted rate throughout the period. Figure 3 presents the results. Studies were not carried out at higher *T*_a_s because in six exploratory tests using the O_2_ measurement protocol at *T*_a_ = 10 °C, nestlings never stopped inhaling and exhaling.

### Studies of O_2_ consumption by apneic nestlings with mouth and nares reversibly occluded

Occlusion was implemented during seven studies of O_2_ consumption at *T*_a_ = 2 °C (not included in Fig. 3) conducted on seven litters (2–3 nestlings per litter). In all cases, the apneic rate of O_2_ consumption was in the typical range for *T*_a_ = 2 °C (Fig. 3) prior to application of occluding wet cotton. However, after occluding cotton was placed over the mouths and nares of the comatose, apneic nestlings, O_2_ consumption was indistinguishable from zero for the period that the cotton was in place. At the end of the O_2_ measurement period, after the cotton was removed and nestlings were placed at *T*_a_ ≅ 20 °C, 10 of the 18 studied individuals (56%) spontaneously resumed ordinary breathing as they rewarmed.

There was an increase in death rate associated with the occlusion experiments. Among the 107 apneic nestlings subjected to ordinary O_2_ consumption measurements (not involving occlusion) at *T*_a_ = 0–2 °C, 100% spontaneously resumed breathing at the end and were known to be alive for at least several days afterward. Among the 18 apneic nestlings subjected to occlusion in the occlusion experiments, the 10 that spontaneously resumed breathing at the end matured to weaning (21 days of age). However, the 8 that failed to resume breathing died. This was true even though, based on close inspection, we concluded that after we removed the wet cotton used for occlusion, the airways of all nestlings were fully open. The observed deaths were scattered among the 7 litters studied. In 6 of the litters (2–3 nestlings per litter), a single individual died while the others lived. In only 1 litter (consisting of 2 individuals) did all individuals die.

The deaths compromised interpretation of the occlusion studies: When we observed a zero rate of O_2_ consumption in a litter of occluded nestlings, the chance existed that some of the individuals were dead or dying rather than merely being occluded. This shortcoming explained why we stopped after study of seven litters.

### Lung morphometrics

**Fig. 4:**
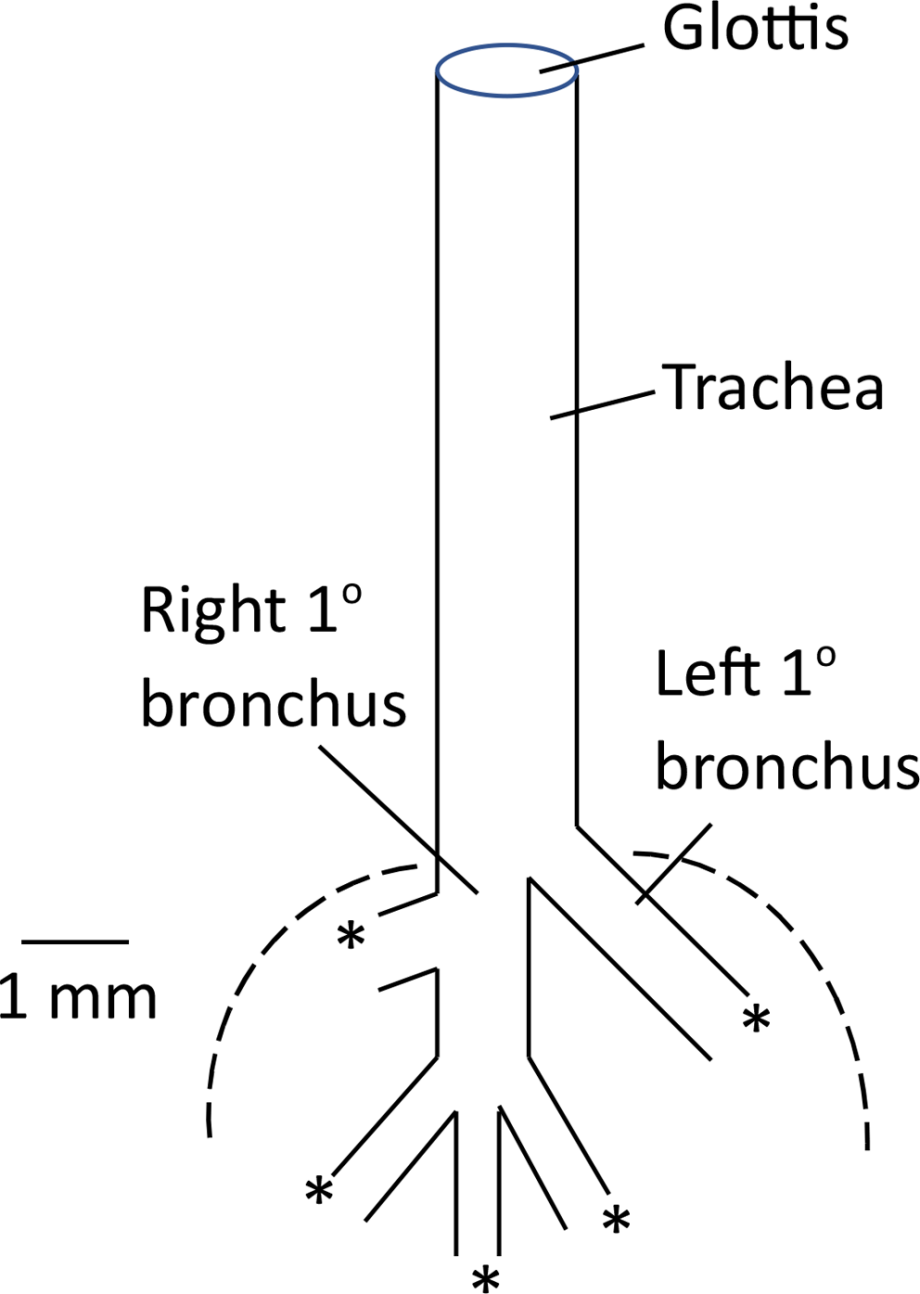
Schematic diagram of major lung airways seen from the ventral side of the body. According to Kling (2011), rodents typically have a left lung composed of one lobe and a right lung composed of 4–5 lobes (see also Metzger et al. 2008). *Peromyscus* adhere to this pattern with 4 lobes in the right lung (Shirkey and Hammond 2014). The parts shown here are scaled correctly relative to each other. The numerical scale applies to mid-ages of the nestling period, ca. 8–13 days of age. Diameters of airways are outside diameters. Asterisks mark spots where the left primary bronchus and the four right secondary bronchi give rise to complex lung parenchyma in the five lung lobes. Dashed lines delineate the approximate position of the anterior margin of the heart (anterior margin of atria) relative to the lung airways. Among the three secondary bronchi at the end of the right primary bronchus, the middle one exhibited notable variation, sometimes being very short or absent

Figure 4 shows the principal lung airways revealed by the dissection we carried out. We measured the lengths and outside diameters of all airways shown (see Supplementary Information). An important point is that the heart is juxtaposed intimately to the airways and parenchyma where shown (anterior margins marked by dashed lines in Fig. 4).

**Fig. 5:**
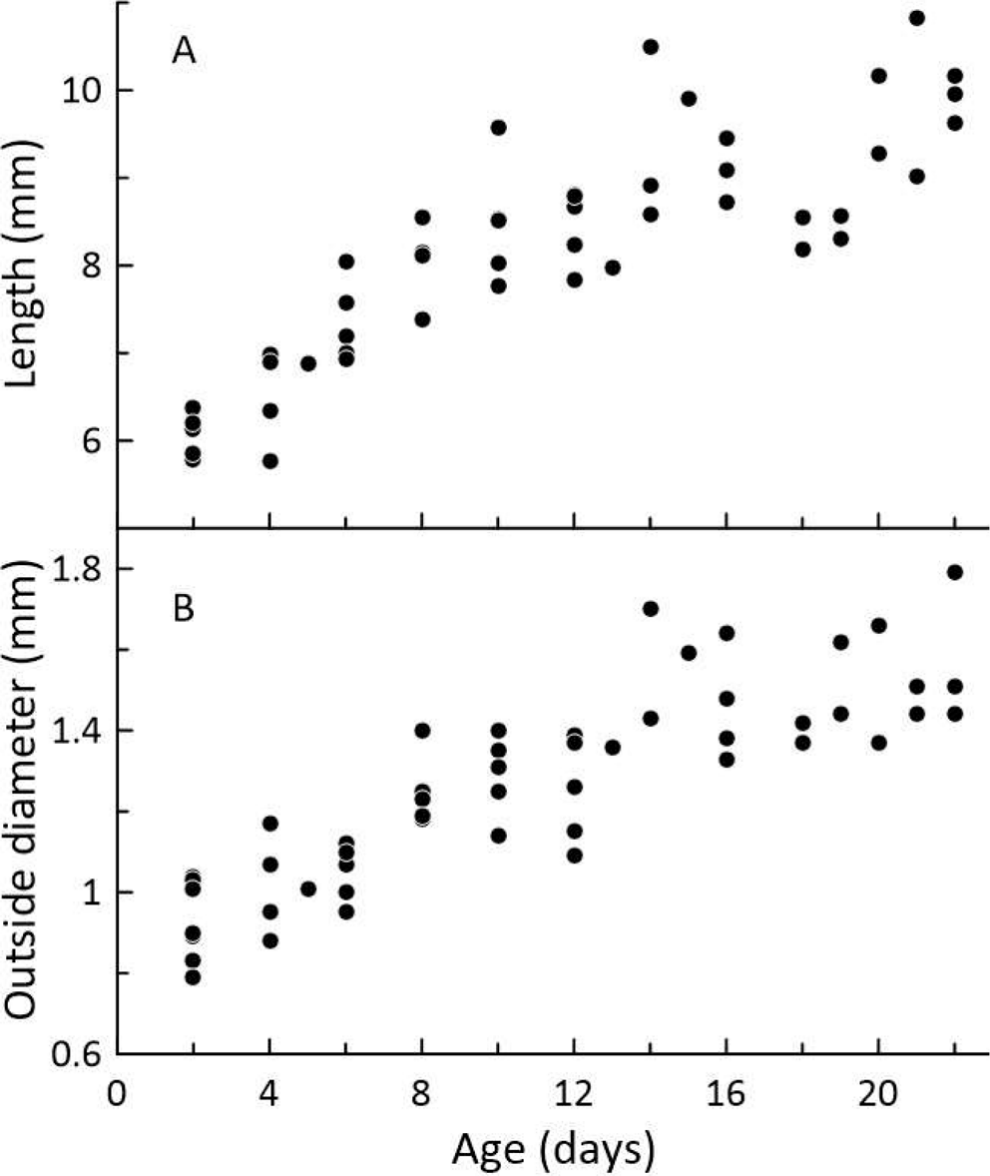
Length (**A**) and outside diameter (**B**) of the trachea as functions of age. Although we gathered data up to age 22 days, our concern in the physiological experiments in this paper is with nestlings aged 11 days and younger. We calculated linear regressions using the data for ages 2–12: *L* = 0.263*a* + 5.66 (*p* < 0.001; r^2^ = 0.80) and *D* = 0.038*a* + 0.860 (*p* < 0.001; r^2^ = 0.62), where *a* is age in days, *D* is outside diameter in mm, and *L* is length in mm. As a check on the linearity of the data in this age range, we used the Segmented package (Muggeo 2008) in R (R Core Team 2021) to test the length data for a discontinuity in slope under a linear model. This procedure identified a discontinuity at age 9 days +/- 1.5 days (SE). Recognizing that the calculated upper bound on the discontinuity age was 10.5 days, we concluded that the regressions are suitable for their intended purpose (see Discussion): assessing the adequacy of tracheal diffusion to supply O_2_ at the rates measured (Fig. 3) in nestlings aged 2–12 days

Tracheal length is shown as a function of age in Fig. 5A. Regarding tracheal outside diameter, duplicate measures were obtained on each individual nestling using the two protocols described in Methods. The results of the duplicate measures were statistically identical, and Fig. 5B shows tracheal outside diameter as a function of age. Tracheal wall thickness did not vary with age and averaged 0.146 mm.

### Electrocardiographic (EKG) studies: nestlings during whole-body exposure to air or N_2_

Lead III (right foreleg and left hindleg) was typically used for EKG recording, although Lead I (right and left forelegs) served as a backup and was occasionally used. In the initial stages of exposure to *T*_a_ = 2–3 °C, as nestlings in air entered hypothermia and their *T*_b_s fell, the amplitude of EKG waveforms declined in parallel. This decline of EKG amplitude in hypothermia explained why we placed exceptional priority on optimal electrical/mechanical shielding at all stages of signal transmission. Noise in our recordings was generally ≤ +/- 2 µV.

After nestlings had been at the target *T*_a_ of 2–3 °C for > 2.3 h and data collection had begun, our beat-by-beat examination of EKG records (see *Data extraction from EKG records*) revealed evidence of switching between two or more ectopic pacing sites (e.g., abrupt changes in the shape of the ventricular waveform) in about 30–40% of the individuals studied in the various parts of the EKG research. These 30–40% were excluded from further analysis for the EKG experiments, for the reasons described earlier, and here we describe results obtained on the remaining nestlings, each of which we judged to evidence an invariant ectopic pacing site.

To test whether whole-body N_2_ exposure exerts effects on cardiac function in apneic nestlings in near-freezing hypothermia, one approach we used was to focus on the 14 nestlings studied in EKG Series I, which were exposed first to air, then N_2_. For each individual we examined the EKG record during the final 16 min of its 30-min exposure to air and the final 16 min of its exposure to N_2_, asking three questions: (1) did the heart rate decrease by ≥ 30% during those 16 min, (2) did the amplitude of the ventricular waveform decrease by ≥ 30% during those 16 min, and (3) did cardiac arrest occur? As seen in Table 1, during exposure to air, none of the studied individuals exhibited changes in rate or amplitude, nor did arrest occur; the nestlings continued heart action in the manner evidenced since the beginning of the experiment. In contrast, during the final 16 min of the subsequent N_2_ interval, all the studied individuals exhibited one or more signs of deteriorating cardiac function.

An important point to note is that the individuals’ responses to N_2_ varied qualitatively; for example, some individuals showed little change in heart rate while exhibiting changes in amplitude, whereas others did the opposite. Under these circumstances, averages calculated for all individuals together would obscure responses. Consider, for example, the individuals showing large heart-rate effects versus those exhibiting no heart-rate effect. Averaging the latter with the former would lead to understatement of the magnitude of the effect in the qualitatively distinct group of individuals for which the principal effect on the heart is a rate effect. Because of these considerations – and to provide future researchers with straightforward knowledge of the type of variability encountered in these studies – we list individual responses in Table 1.

To gain further insight into the effects of whole-body N_2_ exposure, another approach we used was to focus on the 29 nestlings exposed to N_2_, then air in EKG Series I and II, and again we report individual data because of qualitative variation among individuals. As seen in Table 2, with few exceptions, when the apneic, hypothermic nestlings were exposed to a N_2_ atmosphere, they experienced suppression of heart function, and this suppression was fully or partially reversed within 20 min following return to an air atmosphere. During the period that each individual was studied in N_2_, we monitored the EKG continuously and switched to air earlier than planned (30 min) when we judged (from cardiac suppression) that the switch was necessary to avoid lethality. The length of the N_2_ interval thus varied (10–30 min, averaging 20 min). The most common response we observed (Table 2) was for heart rate to remain relatively steady throughout study while amplitude decreased in N_2_ and recovered partly or fully in subsequent air; about half of the individuals displayed this pattern. The second most common response, displayed by about a quarter of individuals, was for both rate and amplitude to decrease in N_2_ and recover partly or fully in air. All nestlings survived, even the one that experienced 24 min of cardiac arrest following N_2_ exposure.

**Table 1:** Effects of whole-body N_2_ exposure on apneic, hypothermic nestlings exposed to air, then N_2_ in EKG Series I: changes in heart rate and amplitude of the ventricular waveform during final 16 min of air interval and during final 16 min of subsequent N_2_ interval. Shown are results for 14 individuals, each of which exhibited a single ectopic pacing site throughout data collection. Heart rate and ventricular amplitude are, respectively, classed as reduced if and only if ≥ 30% lower at the end of the 16-min interval than at the beginning. Cardiac arrest was classed as being present if even a single heartbeat was missed; when a length of time is specified for arrest, no heartbeats occurred for the entire duration. All listed arrests self-terminated with spontaneous return of heart action

Age (days)	Body weight (g)	Effect of air	Effect of *N*_2_
5	3.8	None	Rate reduced; arrest for 5 min
10	4.6	None	Amplitude reduced
5	2.8	None	Rate reduced; arrest for 6 min
7	3.4	None	Amplitude reduced
7	4.5	None	Rate reduced
5	3.4	None	Amplitude reduced
6	3.7	None	Rate and amplitude reduced
12	4.5	None	Rate and amplitude reduced; arrest for 10 min, then another arrest for 14 min
3	2.7	None	Amplitude reduced
4	3.2	None	Rate and amplitude reduced
6	3.6	None	Amplitude reduced
6	3.8	None	Rate and amplitude reduced
8	4.0	None	Rate and amplitude reduced
5	3.0	None	Rate reduced

**Table 2:** Effects of whole-body N_2_ exposure on apneic, hypothermic nestlings: EKG data for 29 individuals exposed first to N_2_, then to air in EKG Series I and II. Data under “Start of N_2_ interval” represent heart rate and amplitude of the ventricular waveform at the very beginning of N_2_ exposure, when the atmosphere in the study chamber had just been switched from air to N_2_. Cardiac arrests (both heart rate and amplitude = 0) occurred in four nestlings; all arrests terminated spontaneously. Each individual included in this table exhibited a single ectopic pacing site throughout data collection

		Start of *N*_2_ interval		End of *N*_2_ interval		Air interval after passage of 20 min	
Age (days)	Body weight (g)	Heart rate (beats/min)	Amplitude (µV)	Heart rate (beats/min)	Amplitude (µV)	Heart rate (beats/min)	Amplitude (µV)
8	5.0	2.8	66	1	33	3.8	64
9	4.5	2.3	46	2.5	18	2	39
7	3.1	4	75	0	0^a^	2	82
7	3.9	4.3	78	2	52	4	57
4	3.2	3.5	47	3.5	27	3.3	42
4	3.2	2.8	45	2.5	19	3	42
5	3.4	3.5	84	1.5	35	3.8	82
6	3.4	3.8	73	1.8	55	3.8	65
6	3.4	4	58	2	25	4	49
3	2.4	2.5	40	3.3	22	3.3	35
6	4.6	4.3	35	1.5	27	3.5	24
5	2.9	3	50	0	0^b^	3	47
9	4.6	3.8	95	3.1	62	3.3	79
9	4.3	3.5	83	3.5	42	3.3	78
9	4.8	2	104	2	50	2	70
9	4.6	2	71	2	26	2	50
10	4.6	2.3	58	2	23	2	65
5	2.8	3.5	75	0	0^c^	2.3	72
7	3.4	3.5	93	2	48	2.3	88
7	4.5	2.8	90	1	78	1	78
5	3.4	3.8	67	4	41	4	58
6	3.7	3.5	52	2.5	15	3.5	54
12	4.5	2	167	0	0^d^	0.8	112
3	2.7	3.3	58	3	26	3	53
4	3.2	2	22	1.3	12	2	15
6	3.6	3.5	59	3.5	20	3.5	50
6	3.8	3.3	63	2.5	32	1.8	58
8	4.0	4.3	56	2	22	2.3	34
5	3.0	3.8	55	2	38	3.5	53

^a, b, c, d^Arrest lasted: *a*, 7 min; *b*, 4 min; *c*, 6 min; *d*, 10 min, then 14 min

### EKG studies: Nestlings during masked studies in which face and body could be exposed to different gases

The manometer between the mask chamber and body chamber, observed throughout these experiments, never registered either static or dynamic pressure differences between the two chambers. The results of the three EKG Series presented in Table 3 point to a dramatic difference between the face and the rest of the body in regards gas exchange with the atmosphere. Specifically, when an N_2_ atmosphere exerts cardiac effects in apneic, hypothermic nestlings, it does so via the mouth and nares (face), not the body at large. Of greater importance, when a nestling in N_2_-induced cardiac suppression is provided with an air atmosphere to enable cardiac recovery, the nestling’s effective gas exchange with the air is principally or exclusively via the mouth and nares. During the induction of N_2_-induced cardiac suppression (caused by N_2_ in the mask chamber), the provision of air around the body posterior to the face does not prevent the cardiac suppression, but when air is thereafter provided to the face, recovery occurs. In fact, this recovery when the face is in air occurs even when the rest of the body is in N_2_.

Among the experiments in Table 3, cardiac arrests occurred only in nestlings subjected to EKG Series IV and V. In cases in which arrests occurred in these Series, the arrests consistently appeared (only one exception) after the start of Step B in the protocols; that is, they occurred following relatively full development of N_2_-induced cardiac suppression. Among nestlings that spontaneously resumed heart action following arrests during the study period (some of which experienced 2–3 arrests interrupted by periods of heart action), arrests lasted 3–21 min (average: 6 min). In two of the three arrested nestlings that remained in arrest until the end of the study period, arrests lasted 25–30 min, but the nestlings nonetheless lived by spontaneously recovering after the study period.

EKG Series VI, carried out on masked nestlings, was a three-step protocol designed to examine the responses of single individuals to both face-only N_2_ and body-only N_2_ within a short span of time, under test conditions that did not substantially suppress heart action prior to either test. In each of the 14 nestlings studied, when the face alone was exposed to N_2_, cardiac suppression occurred (i.e., ≥ 30% reduction in rate, amplitude, or both, followed in 2 cases by arrest). However, when the same nestlings had earlier been subjected to body-only N_2_, most (13) had shown no response (although one exhibited ambiguous hints of an effect).

**Table 3:** Results of EKG studies on masked, apneic, hypothermic nestlings (3–11 days old) subjected to three two-step protocols: EKG Series III, IV, and V. Each trial was carried out on a different nestling. In all cases, nestlings had been living continuously in air prior to step A. In this table “suppression” means a ≥ 30% reduction in amplitude, rate, or both. Recovery could be full or partial. No cardiac arrests occurred in Series III. Each studied individual exhibited a single ectopic pacing site throughout data collection

Evidence for a negative effect of *N*_2_ on heart action	Number of trials producing type of evidence specified		
	Series III: Body exposed to N_2_ in Step A, then air in Step B; face exposed to air continuously (total number of trials: 22)	Series IV: Face exposed to N_2_ in Step A, then air in Step B; body exposed to air continuously (total number of trials: 5)	Series V: Face exposed to N_2,_ body to air, in Step A, then face exposed to air, body to N_2_ in Step B (total number of trials: 18)
No evidence^a^	17	0	0
Ambiguous evidence^b^	3	0	1
Firm evidence: suppression followed by recovery	2	4	10
Firm evidence: suppression and arrest(s), followed by recovery	0	1	4
Firm evidence: suppression followed by an arrest that lasted to end of study period	0	0	3

^a^ Rate and amplitude steady for entire study period with only routine variation (usual), or rate and amplitude underwent moderate, steady decline from the start to the finish of the entire study period

^b^Hints of effects, but EKG changes small, variation great, or uncertain synchrony between EKG changes and gas changes

## Discussion

Nestling white-footed mice (*Peromyscus leucopus*) are born in the earliest days of spring in cold climates such as found in Michigan and Ontario (Long 1973; Millar et al. 1979; Millar and Gyug 1981). If early-age nestlings are by accident exposed to near-freezing temperatures, e.g., *T*_a_ = 0–5 °C, they may experience *T*_b_s equally low. They have an impressive ability to tolerate such near-freezing hypothermia even though they become apneic (Hill 2017). We show that nestlings (2–10 days old) in this state of apnea undergo physiologically important exchanges of gases with the atmosphere. These gas exchanges do not occur across the skin. Instead they occur via the trachea and lungs even though the animals are not inhaling or exhaling. Most significantly, when neonates are in apnea in ordinary air, they take up O_2_ steadily from the atmosphere throughout the apneic period, and the evidence available indicates that this O_2_ uptake is essential for the nestlings’ survival of the apneic period. Neonatal tolerance of near-freezing hypothermia is not universal. For example, the lowest *T*_b_s that early-age nestlings of feral and inbred *Mus musculus* (house mice) can survive are about 5 °C higher than those *P. leucopus* nestlings tolerate (Hill 2017). The exceptional physiology of *P. leucopus* nestlings at near-freezing temperatures seems likely to be an important factor in the species’ success in cold climates, where, for example, white-footed mice prosper in the wide expanses of the natural forests, whereas house mice are largely restricted to protective human-made microhabitats like houses and outbuildings (Kurta 2017).

In the mid-20th century, understanding of hypothermic rodent nestlings was impeded by a critical error: a conviction that nestlings in deep hypothermia are in continuous cardiac arrest (Hill 2000). Although the error was undoubtedly innocent and straightforward explanations for it are easy to postulate (Hill 2000), the error remained unrecognized for several decades, and the prominent physiologist E.F. Adolph, who published original research on the subject (Adolph 1951, 1963), gave prominence to “biological zero” states (e.g., zero heart rate) in his influential book, Adolph (1968). Little reason existed to wonder about pulmonary O_2_ uptake when the blood was considered to be immobile (and thus unable to deliver O_2_ to systemic tissues). A zero state in ventilation – apnea – was recognized in deeply hypothermic nestlings but assumed to represent zero pulmonary O_2_ uptake, which was viewed simply as a logical corollary of the zero state in heart action – cardiac arrest.

For us, the reversal of these earlier misunderstandings was driven by EKG studies of the heart and circulation, and those studies came first. By accident, in the 1990s Hill (2000) discovered by means of EKG studies on nestlings of several rodent species (including the species Adolph and colleagues studied) that the heart continues to beat throughout periods of apneic hypothermia that the nestlings are able to survive. This discovery set the stage (see Introduction) for another accident, which arose during later EKG studies: our discovery, reported here, that a N_2_ atmosphere affects heart action in hypothermic nestlings that are not inhaling and exhaling.

Our overarching hypothesis at present is that beating of the heart and circulation of the blood during near-freezing hypothermia are essential for survival of the hypothermia because the circulation delivers O_2_, which is taken up by the apneic lungs. Because we ended our EKG experiments whenever their continuance posed a threat to the lives of nestling subjects, we do not have unambiguous evidence that heart action and circulation are essential for survival in deep hypothermia. However, as we conducted dozens of EKG experiments on deeply hypothermic nestlings in air, we developed a keen sense that if a spontaneous cardiac arrest lasted ≥ 30 min, the nestling would likely die unless rewarmed immediately. As our data (e.g., Tables 1 and 2) show, *P. leucopus* nestlings reliably tolerate self-terminating cardiac arrests that last up to 15–20 min. Cardiac arrests in hypothermia, that is, do not kill quickly (presumably because of the profound metabolic depression at near-freezing *T*_b_s). However, our hypothesis is that arrests ultimately kill and do so because of the circulatory system’s failure to deliver O_2_ when the heart is in arrest.

When apneic *P. leucopus* nestlings in near-freezing hypothermia are placed in a N_2_ atmosphere, the consequences for heart action and the EKG are deterministic in the sense that one or more negative impacts are virtually guaranteed to occur: Heart rate declines, electrical amplitude of the ventricular waveform decreases, and/or cardiac arrests are precipitated (Tables 1, 2 and 3). The decline in heart rate and the arrests are clearly negative impacts. Although the significance of a decrease in EKG electrical amplitude is not entirely unambiguous, we interpret the reduced amplitude as a negative development because, in hypothermic *P. leucopus* nestlings, it is consistently associated with other signs of cardiac deterioration.

The physiological effects of atmospheric N_2_ on apneic nestlings have three highly significant attributes. First, the effects are reversible if the N_2_ atmosphere is replaced with air (Table 2). Second, the effects are mediated via the lungs, demonstrated by the fact that in masked nestlings, N_2_ effects occur if only the face (including nares and mouth) is exposed to N_2_ but do not occur if only the rest of the body is exposed to N_2_ (Table 3). Third, the EKG effects of atmospheric N_2_ on apneic nestlings start to occur rapidly, often within 5–10 min of the imposition of the N_2_ atmosphere, implying that – despite apnea – easy gas exchange takes place between atmosphere, lungs, and blood flowing in the coronary circulation.

When we refer to apneic *P. leucopus* nestlings, it is important to recognize the evidence we have that the nestlings are in fact not inhaling and exhaling. As summarized in Results, we have abundant visual, electrical, and auditory evidence that nestling *P. leucopus* that have reached steady-state in near-freezing hypothermia are truly apneic. Pulmonary physiologists (Tattersall and Milsom 2003; Corcoran et al. 2012) have carried out thorough studies on nestlings of other rodent species, e.g., lab rats (*Rattus norvegicus*) and Syrian hamsters (*Mesocricetus auratus*), and have also obtained convincing evidence for true apnea in hypothermia.

**Fig. 6:**
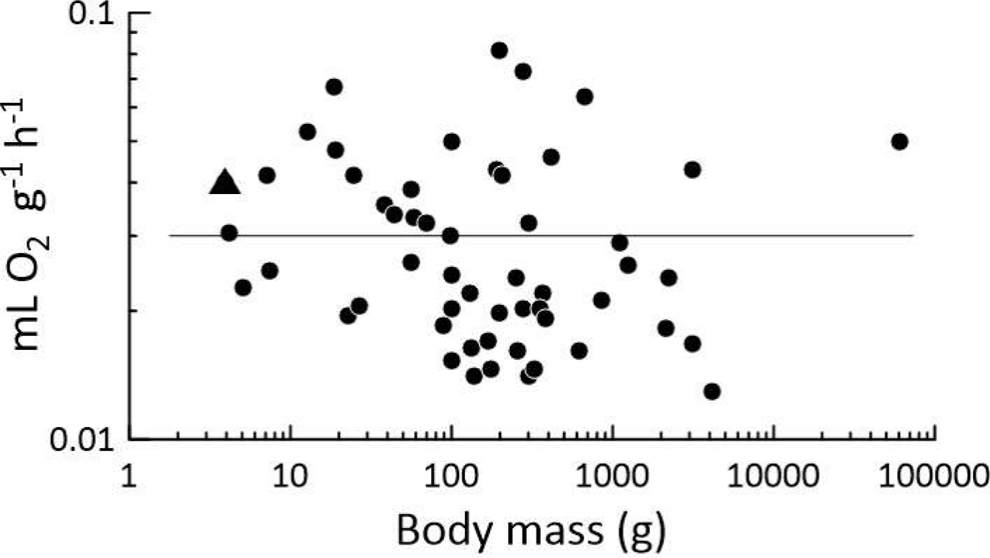
Average rate of O_2_ consumption by hypothermic, apneic *P. leucopus* nestlings relative to rates observed in hibernating adult mammals. The overall plot is replicated from Heldmaier et al. (2004), who summarized data (circular symbols) from > 50 studies on adults in hibernation at *T*_a_s ranging from 2 °C to 7 °C. The line is the regression between rate of O_2_ consumption and body mass calculated by Heldmaier et al. (2004), indicating that the mean rate of O_2_ consumption of hibernators in hibernation is 0.03 mL O_2_ g^− 1^ h^− 1^, independent of body mass. The triangle depicts the average rate of O_2_ consumption (Fig. 3) of apneic, hypothermic *P. leucopus* nestlings at similar *T*_a_s (and presumptive *T*_b_s)

After EKG studies convinced us (Tables 1, 2 and 3) that, despite apnea, hypothermic *P. leucopus* nestlings dynamically exchange gases with the atmosphere via their lungs, we undertook our direct studies of O_2_ consumption. These studies directly demonstrate that apneic, deeply hypothermic nestlings take up O_2_ steadily from the atmosphere at a rate that varies quasi-exponentially with *T*_b_ (Fig. 3). In 2004, Heldmaier et al. (2004) published an update of an earlier hibernation review (Heldmaier and Ruf 1992), in which they confirmed their revelation that – based on their approach to the analysis – the rate of O_2_ consumption (mL O_2_ g^− 1^ h^− 1^) of adult mammalian hibernators when in hibernation is independent of body mass over a range of 5 orders of magnitude of mass (see also Singer and Mühlfeld 2007). Heldmaier et al. (2004) specifically summarized data on hibernators studied at *T*_a_ = 2–7 °C, conditions under which *T*_b_ ≅ 2–7 °C in species of small to moderate body size. When we average the mean rates of O_2_ consumption by hypothermic, nestling *P. leucopus* at similar *T*_a_s (i.e., 2 °C, 4 °C, and 7 °C; Fig. 3), we obtain an overall mean of 0.04 mL O_2_ g^− 1^ h^− 1^. Thus, as seen in Fig. 6, these nestlings, although apneic, not only consume O_2_ but do so at rates in the identical range as adult mammals in hibernation at similar *T*_a_s (Heldmaier et al. 2004). A decade after the analysis by Heldmaier et al. (2004), Ruf and Geiser (2015) published a fresh analysis of the rate of O_2_ consumption of adult mammalian hibernators in hibernation (see also Geiser 2004). Possibly because they (unlike Heldmaier et al. 2004) used a phylogenetically informed statistical model, they obtained evidence that, among species of adult hibernators, the hibernation metabolic rate (mL O_2_ g^− 1^ h^− 1^) decreases “slightly” (their word choice) as body mass increases. For the hibernators included in their analysis, they reported a grand mean hibernation metabolic rate of 0.04 mL O_2_ g^− 1^ h^− 1^ (95% confidence limits: 0.036–0.043 mL O_2_ g^− 1^ h^− 1^), and their regression of hibernation metabolic rate against body weight predicts a rate of about 0.05 mL O_2_ g^− 1^ h^− 1^ for a hibernator of similar body weight to a 6-day-old *Peromyscus* nestling, supporting the conclusion that deeply hypothermic *P. leucopus* nestlings consume O_2_ at rates similar to adult hibernators in hibernation. As to the site of O_2_ uptake by apneic, hypothermic *P. leucopus* nestlings, just as our EKG studies of masked nestlings in a N_2_ atmosphere point to the lungs (Table 3), our studies of nestling O_2_ consumption before and after facial occlusion (despite significant shortcomings) also point to the lungs.

The final topic we address in this report is the physiology of gas transport in the lungs. A few prior papers have been published on mechanisms of apneic pulmonary gas exchange in rodents. We have turned to these papers for insight, although all concern, not neonates, but adult hibernators. The papers focus on the transient, self-terminating intervals of apnea that routinely occur during adult hibernation in some hibernating species (Milsom and Jackson 2011). Three papers use mechanistic equations to quantify apneic pulmonary gas exchange: Szewczak and Jackson (1992) studying bats, Sullivan and Szewczak (1998) studying pocket mice, and Wilz et al. (2000) studying dormice. The challenges of such quantification are great, and because of the challenges, only diffusion is quantified in the three papers, using similar versions of the diffusion equation.

A first point to stress is that, as summarized by Szewczak and Jackson (1992), there are three potential mechanisms by which the lungs of an apneic mammal can obtain O_2_ for uptake into the blood: (1) simple diffusion of O_2_ from the atmosphere to the lungs along the length of the trachea and other airways; (2) unidirectional mass flow of air from the atmosphere into the lungs through the airways, arising from the fact that, whenever the respiratory quotient (RQ) is < 1, the volume of O_2_ removed from the lung gas by metabolism is not volumetrically fully replaced with CO_2_, resulting in a sucking force; and (3) bellows-like mass flow of air from the atmosphere toward the lungs, caused by the fact that the heart is closely juxtaposed to lung airways (Fig. 4), which are therefore alternately compressed and relaxed as the heart beats. The third mechanism, often termed “cardiogenic mixing,” is aptly described by Szewczak and Jackson (1992) as a process that accelerates diffusion by inducing mechanical agitation in the airways.

Focusing on apneic, hypothermic 10-day-old *P. leucopus*, we have followed the procedure of Wilz et al. (2000) to calculate a quantitative estimate of pulmonary O_2_ uptake by diffusion.^5^ Based on the regressions in the legend of Fig. 5, the mean tracheal length of 10-day-olds is 8.29 mm, and the mean outside tracheal diameter is 1.24 mm. We estimate tracheal lumen diameter to be 0.95 mm by subtracting twice our measured wall thickness (0.146 mm) from outside diameter. With these dimension measures, the equation of Wilz et al. (2000), which assumes atmospheric O_2_ partial pressure at the glottis, estimates O_2_ uptake by diffusion in an apneic 10-day-old *P. leucopus* to be 0.66 mL O_2_ h^− 1^, or (given that 10-day-olds in the present study weighed an average of 5.2 g) about 0.13 mL O_2_ g^− 1^ h^− 1^. By this method of estimation, therefore, gas transport by diffusion alone can readily supply deeply hypothermic 10-day-olds with O_2_ at the rates the nestlings consume O_2_ (Fig. 3) at all *T*_a_s studied. The same conclusion applies to other ages. Calculated by the method of Wilz et al. (2000), O_2_ uptake by diffusion (mL O_2_ h^− 1^) in 2-day-olds is about 0.6 times that in 10-day olds based on the differences in tracheal dimensions (Fig. 5). Two-day-olds weigh only about half as much as 10-day-olds (Hill 1976), however, so weight-specific O_2_ delivery is about the same.

Of course, as explicitly stated in all the papers we cite on apneic O_2_ uptake, an approach like that of Wilz et al. (2000) quantifies only the mechanism of apneic pulmonary O_2_ uptake that is simplest to quantify: diffusion through a simple tube (the trachea). The dimensions of the gas-exchange pathway from atmosphere to glottis are not considered by Wilz et al. (2000) and have never been quantified (or even described qualitatively) in any rodent species. In each individual *P. leucopus* nestling studied for Fig. 5, we measured the linear distance from glottis to the anterior surface of the muzzle (see Supplementary Information). In 10-day-olds, this distance is 1.1–1.2 cm. Even if we guess that the cross-sectional area of the gas-exchange pathway from atmosphere to glottis is merely the same as the tiny cross-sectional area of the tracheal lumen (a conservative guess, but the one already adopted by Sullivan and Szewczak 1998), we would estimate O_2_ uptake by diffusion in 10-day-old *P. leucopus* from atmosphere to lungs, using the equation of Wilz et al. (2000), to be about 0.05 mL g^− 1^ h^− 1^, still an adequate rate to meet measured O_2_ consumption in deep hypothermia (Fig. 3), at least at *T*_b_s of 4 °C or lower. Emphatically, however, the diffusion calculations entirely omit the effects of the other two potential mechanisms of apneic O_2_ transport that might be operative in nestling *P. leucopus* lungs (RQ-driven mass flow and cardiogenic mixing), both of which increase O_2_ delivery to rates above the diffusion-driven rate. Recently, Dubsky et al. (2018) have greatly enhanced understanding of cardiogenic mixing. Studying adult lab mice with synchrotron-based dynamic imaging, they were able for the first time to quantify the effects of cardiogenic mixing. They found that cardiogenic mixing can potentially be of enormous significance during apnea because it can cause as much as a four-fold increase in gas mixing in the pulmonary airways.

All things considered, while acknowledging the considerable ignorance that remains, it seems quite plausible to conclude that the known mechanisms available to drive O_2_ transport from the atmosphere to the lungs during apnea in hypothermic nestling *P. leucopus* are sufficient to meet the nestlings’ measured O_2_ needs (Fig. 3). No doubt, the analysis possible at this time is more suggestive than definitive, as also noted by Szewczak and Jackson (1992), Sullivan and Szewczak (1998), and Wilz et al. (2000). Nonetheless, this analysis at least has the virtue of clarifying the questions to be addressed by future research.

An important concluding point is to emphasize the three-fold significance of the continued beating of the heart during apneic hypothermia in *P. leucopus*. First, without the heart beat, there would be no cardiogenic mixing, whereas in fact, because of the heart’s position (Fig. 4), the beating of the heart likely contributes significantly to pulmonary O_2_ uptake (Dubsky et al. 2018). Second and most obviously, the beating of the heart circulates the blood, without which O_2_ uptake by the lungs would be essentially pointless. Third, whether we consider ordinary diffusion or cardiogenically aided diffusion, the circulation of the blood is instrumental in maintaining a low enough O_2_ partial pressure in the lungs to drive the necessary inward O_2_ diffusion through the trachea and other pulmonary airways.

## Electronic supplementary material

Below is the link to the electronic supplementary material.

Supplementary Material 1

## Data Availability

All measurements of oxygen consumption and pulmonary morphometrics are included in this publication and the supplementary tables of this publication. Original electrocardiograms were physically recorded, and those records (which consist of 100s of pages) are available from the Corresponding Author.

## Footnotes

1 A future paper, focused on comparative evidence from multiple rodent species, will discuss this phenomenon in detail.

2 Because of the low mass-specific rates of O_2_ consumption by deeply hypothermic nestlings, the percentage of O_2_ in the air contained inside the sealed respirometry system did not fall below 20% even when relatively heavy nestlings were studied for the maximum duration (2.5 h).

3 The pressure-stabilizing device, constructed with a 3-mL syringe, could alter the total volume during an experiment by about 2%, introducing a small ambiguity.

4 When nestlings aged 4–10 days were removed from animal-colony conditions and placed alone at *T*_a_ = 2–3 °C, apnea set in after 43–72 min (mean: 66 min) according to electromyographic signatures in EKG records. After apneic nestlings had been held at *T*_a_ = 2–3° for several hours and were suddenly placed at *T*_a_ ≅ 20 °C, nestlings aged 3–6 days spontaneously terminated apnea (started to inhale and exhale) after 9–17 min (mean: 12 min), and nestlings aged 7–10 days started after 13–22 min (mean: 15 min).

5 This procedure rests on assumed values (itemized by Wilz et al. 2000) for some physiological variables.
