# Supplementary material for: Apneic uptake of atmospheric O2 by deeply hypothermic nestlings of the white-footed mouse (Peromyscus leucopus): circulation and lungs
Source: J Comp Physiol B. 2024 Oct 8;195(1):123–39. doi: 10.1007/s00360-024-01585-x (PMC11839856; doi:10.1007/s00360-024-01585-x)
Supplement: Supplementary file 1 — Supplementary Material 1 [file 360_2024_1585_MOESM1_ESM.docx]

**Supplementary Information**

Presented here are the morphometric data gathered on the lungs of nestlings as described in the paper. The diagram below identifies the airways that were visible in our dissection and, in the case of each airway, shows diagrammatically the length of the airway that was visible and measured. The line inside the right 1° bronchus defines the length measured for that airway. Note in the tables of data that background data for each studied individual included body length and weight as well as age. Blanks are entered in the tables where data are missing. Nestling numbers are arbitrary but are consistent between the two tables of data. All measures are in mm unless stated otherwise in the column headings. “L” means length. “D” means outside diameter. Body length is the distance between tip of nose and base of tail. Mouth length is distance between anterior larynx and tip of muzzle. In the tables, LPB = left primary bronchus, RPB = right primary bronchus; 2a, 2b, and 2c refer to the secondary bronchi shown in the diagram.

2^o^*c*

2^o^*b a* ®®®

2^o^*a a* ®®®

Right 1^o^
bronchus
bronchus

Glottis

Left 1^o^
bronchus

Trachea

| **Nestling** | **Age (days)** | **Sex** | **BW (g)** | **Body  L (cm)** | **Mouth L (cm)** | **Trachea L** | **Trachea D** | **LPB L** | **LPB D** | **RPB L** | **RPB D** |
| --- | --- | --- | --- | --- | --- | --- | --- | --- | --- | --- | --- |
| 1 | 21 | M | 10.43 | 7.1 |  | 9.02 | 1.44 |  |  |  |  |
| 2 | 14 | M | 6.24 | 6.2 |  | 8.59 |  |  |  |  |  |
| 3 | 22 | F | 10.85 | 7.8 |  | 9.96 | 1.44 | 3.32 | 0.76 | 1.83 | 0.96 |
| 4 | 12 | M | 5.74 | 5.9 | 1.25 | 7.84 | 1.15 | 2.11 | 0.58 | 1.86 | 0.87 |
| 5 | 13 | F | 5.93 | 6.1 | 1.20 | 7.97 | 1.36 | 3.42 | 0.67 | 2.14 | 0.79 |
| 6 | 6 | M | 4.05 | 4.9 | 1.05 | 7.20 | 1.07 | 2.49 | 0.69 | 1.74 | 0.91 |
| 7 | 8 | F | 5.55 | 5.5 | 1.15 | 8.55 | 1.40 | 3.19 | 0.76 | 2.50 | 1.08 |
| 8 | 16 | M | 6.52 | 6.5 | 1.20 | 9.08 | 1.33 | 3.25 | 0.67 | 1.66 | 0.87 |
| 9 | 12 | F | 4.71 | 5.4 | 1.05 | 8.67 | 1.26 | 2.91 | 0.63 | 2.07 | 0.84 |
| 10 | 10 | M | 6.64 | 6.1 | 1.20 | 8.53 | 1.40 | 2.58 | 0.70 | 1.84 | 0.99 |
| 11 | 4 | F | 3.43 | 4.8 | 1.00 | 6.98 | 1.07 | 2.67 | 0.70 | 1.78 | 0.77 |
| 12 | 4 | F | 2.51 | 4.1 | 0.90 | 5.76 | 1.17 | 2.50 | 0.52 | 1.47 | 0.89 |
| 13 | 6 | F | 4.30 | 5.1 | 1.10 | 8.04 | 1.12 | 2.39 | 0.64 | 1.82 | 0.84 |
| 14 | 20 | M | 8.94 | 7.2 | 1.30 | 9.27 | 1.66 | 3.50 | 0.91 | 2.05 | 1.27 |
| 15 | 2 | M | 2.59 | 4.4 | 0.90 | 6.15 | 1.04 | 2.23 | 0.52 | 1.94 | 0.76 |
| 16 | 2 | M | 2.48 | 4.2 | 0.90 | 6.36 | 1.03 | 2.66 | 0.63 | 1.81 | 0.79 |
| 17 | 8 | M | 4.63 | 5.2 | 1.13 | 8.11 | 1.25 | 2.57 | 0.74 | 1.84 | 1.05 |
| 18 | 2 | F | 2.60 | 4.1 | 0.90 | 6.38 | 1.01 | 1.99 | 0.54 | 1.51 | 0.85 |
| 19 | 16 | F | 7.39 | 6.9 | 1.30 | 8.73 | 1.38 | 2.40 | 0.66 | 2.19 | 0.98 |
| 20 | 18 | F | 7.72 | 7.0 | 1.25 | 8.55 | 1.37 | 2.65 | 0.85 | 2.20 | 1.25 |
| 21 | 22 | F | 12.23 | 7.8 | 1.28 | 9.63 | 1.79 | 3.45 | 0.75 | 2.81 | 1.12 |
| 22 | 19 | M | 10.07 | 7.3 | 1.30 | 8.31 | 1.44 | 3.38 | 0.71 | 2.77 | 1.15 |
| 23 | 20 | M | 8.40 | 6.9 | 1.30 | 10.17 | 1.37 | 4.04 | 0.94 | 2.53 | 1.18 |
| 24 | 22 | M | 10.65 | 7.6 | 1.35 | 10.17 | 1.51 | 3.49 | 0.51 | 2.92 | 0.88 |
| 25 | 18 | F | 7.02 | 7.0 | 1.30 | 8.18 | 1.42 | 3.11 | 0.68 | 2.67 | 1.06 |
| 26 | 21 | F | 10.90 | 7.5 | 1.30 | 10.82 | 1.51 | 2.34 | 0.71 | 1.67 | 1.11 |
| 27 | 12 | M | 6.04 | 6.3 | 1.20 | 8.81 | 1.39 | 2.18 | 0.55 | 1.81 | 1.09 |
| 28 | 19 | M | 7.07 | 6.9 | 1.30 | 8.56 | 1.62 | 2.75 | 0.86 | 2.02 | 1.16 |
| 29 | 14 | M | 7.32 | 6.6 | 1.30 | 8.91 | 1.70 | 2.52 | 0.73 | 2.23 | 1.20 |
| 30 | 2 | F | 2.64 | 4.0 | 0.90 | 5.78 | 0.83 | 2.27 | 0.55 | 1.50 | 0.70 |
| 31 | 16 | M | 7.66 | 6.7 | 1.25 | 8.72 | 1.64 | 3.11 | 0.86 | 2.98 | 1.10 |
| 32 | 2 | M | 2.42 | 4.1 | 0.85 | 6.14 | 0.89 | 2.08 | 0.56 | 1.72 | 0.72 |
| 33 | 2 | M | 2.37 | 4.0 | 0.87 | 6.21 | 0.79 | 2.01 | 0.51 | 1.34 | 0.68 |
| 34 | 4 | M | 3.33 | 4.8 | 0.97 | 6.89 | 0.95 | 2.12 | 0.56 | 1.18 | 0.92 |
| 35 | 4 | M | 3.82 | 4.7 | 1.05 | 6.34 | 0.88 | 1.81 | 0.50 | 1.57 | 0.74 |
| 36 | 2 | F | 2.04 | 3.8 | 0.80 | 5.86 | 0.90 | 1.57 | 0.52 | 1.28 | 0.69 |
| 37 | 6 | F | 3.88 | 4.8 | 1.04 | 7.01 | 0.95 | 1.81 | 0.52 | 1.34 | 0.92 |
| 38 | 8 | M | 4.46 | 5.1 | 1.15 | 7.39 | 1.18 | 2.27 | 0.66 | 1.46 | 0.91 |
| 39 | 6 | M | 3.89 | 4.8 | 1.04 | 6.94 | 1.10 | 2.09 | 0.71 | 1.88 | 0.88 |
| 40 | 8 | F | 4.82 | 5.3 | 1.10 | 8.15 | 1.23 | 2.29 | 0.63 | 1.67 | 0.89 |
| 41 | 6 | M | 3.57 | 4.6 | 1.00 | 7.58 | 1.00 | 2.15 | 0.54 | 1.48 | 0.68 |
| 42 | 10 | F | 5.87 | 5.9 | 1.25 | 8.02 | 1.35 | 2.46 | 0.84 | 1.88 | 1.21 |
| 43 | 5 | F | 3.81 | 4.8 | 1.06 | 6.88 | 1.01 | 2.11 | 0.61 | 1.55 | 1.02 |
| 44 | 8 | F | 4.71 | 5.3 | 1.17 | 8.11 | 1.19 | 2.20 | 0.76 | 1.44 | 1.03 |
| 45 | 10 | M | 5.57 | 5.7 | 1.16 | 8.52 | 1.25 | 2.67 | 0.65 | 1.97 | 0.98 |
| 46 | 12 | F | 5.51 | 5.6 | 1.17 | 8.24 | 1.09 | 2.68 | 0.68 | 2.22 | 0.81 |
| 47 | 10 | F | 4.23 | 5.2 | 1.10 | 7.77 | 1.14 | 2.62 | 0.59 | 1.72 | 0.94 |
| 48 | 16 | F | 8.17 | 7.0 | 1.35 | 9.45 | 1.48 | 2.57 | 0.77 | 2.14 | 1.02 |
| 49 | 10 | M | 5.79 | 5.9 | 1.20 | 9.58 | 1.31 | 2.50 | 0.68 | 1.70 | 1.00 |
| 50 | 15 | F | 8.97 | 7.1 | 1.38 | 9.91 | 1.59 | 3.20 | 0.80 | 2.16 | 1.32 |
| 51 | 14 | F | 7.72 | 6.5 | 1.25 | 10.50 | 1.43 | 2.83 | 0.84 | 2.33 | 0.98 |
| 52 | 12 | M | 6.59 | 6.1 | 1.25 | 8.79 | 1.37 | 2.63 | 0.62 | 1.70 | 0.93 |

| **Nestling** | **2c L** | **2b L** | **2a L** | **2c D** | **2b D** | **2a D** |
| --- | --- | --- | --- | --- | --- | --- |
| 1 |  |  |  |  |  |  |
| 2 |  |  |  |  |  |  |
| 3 | 1.54 | 0.80 | 0.39 | 0.66 |  | 0.74 |
| 4 | 1.13 | 1.58 | 0.66 | 0.39 | 0.42 | 0.78 |
| 5 | 1.49 | 0.79 | 0.76 | 0.49 | 0.63 | 0.73 |
| 6 | 1.08 | 0.95 | 0.93 | 0.58 | 0.47 | 0.81 |
| 7 | 1.90 | 1.83 | 0.97 | 0.72 | 0.57 | 0.73 |
| 8 | 1.65 | 1.42 | 0.66 | 0.47 | 0.43 | 0.68 |
| 9 | 1.46 | 1.66 | 0.51 | 0.48 | 0.36 | 0.64 |
| 10 | 1.40 | 1.81 | 0.72 | 0.46 | 0.54 | 0.75 |
| 11 | 1.39 | 0.85 | 0.63 | 0.53 | 0.44 | 0.82 |
| 12 | 1.56 |  | 0.73 | 0.59 |  | 0.65 |
| 13 | 1.34 | 1.66 | 0.76 | 0.55 | 0.43 | 0.69 |
| 14 | 2.45 | 1.73 | 0.95 | 0.72 | 0.60 | 1.07 |
| 15 | 1.30 | 1.41 | 0.62 | 0.48 |  | 0.62 |
| 16 | 1.35 | 1.12 | 0.67 | 0.39 | 0.45 | 0.68 |
| 17 | 1.77 | 1.38 | 0.60 | 0.64 | 0.60 | 0.84 |
| 18 | 1.24 | 0.61 | 0.32 | 0.44 | 0.37 | 0.58 |
| 19 | 1.63 | 1.28 | 0.75 | 0.50 | 0.36 | 0.88 |
| 20 | 2.35 | 1.05 | 0.63 | 0.75 | 0.55 |  |
| 21 | 2.12 | 0.86 | 0.61 |  |  | 1.09 |
| 22 | 2.15 | 1.48 | 0.94 | 0.59 | 0.54 | 1.04 |
| 23 | 2.40 | 1.74 | 0.87 | 0.69 | 0.81 | 1.07 |
| 24 | 2.26 | 1.51 | 0.57 | 0.54 | 0.61 | 1.01 |
| 25 | 1.44 | 1.55 | 0.68 | 0.56 | 0.53 | 0.97 |
| 26 | 2.21 | 0.99 | 0.73 | 0.52 | 0.54 | 0.96 |
| 27 | 1.54 | 1.88 | 0.68 | 0.56 | 0.38 | 0.91 |
| 28 | 1.33 | 1.73 | 0.57 | 0.46 | 0.43 | 1.13 |
| 29 | 1.44 | 1.43 | 0.57 | 0.64 | 0.53 | 0.87 |
| 30 | 0.95 | 0.66 | 0.32 | 0.46 | 0.48 | 0.62 |
| 31 | 1.97 | 1.08 | 0.60 | 0.54 | 0.53 | 0.99 |
| 32 | 1.35 | 1.22 | 0.56 | 0.47 | 0.49 | 0.62 |
| 33 | 1.03 | 0.93 | 0.43 | 0.43 | 0.43 | 0.50 |
| 34 | 1.25 | 1.54 | 0.34 | 0.56 | 0.47 | 0.65 |
| 35 | 1.31 | 1.27 | 0.40 | 0.51 | 0.50 | 0.57 |
| 36 | 1.02 | 1.10 | 0.34 | 0.40 | 0.42 | 0.53 |
| 37 | 1.02 | 1.13 | 0.31 | 0.53 | 0.46 | 0.66 |
| 38 | 1.46 | 1.79 | 0.78 | 0.51 | 0.50 | 0.82 |
| 39 | 1.30 | 1.14 | 0.79 | 0.57 | 0.48 | 0.72 |
| 40 | 1.46 | 1.33 | 0.68 | 0.44 |  | 0.77 |
| 41 | 1.19 | 1.11 | 0.60 | 0.46 | 0.43 | 0.68 |
| 42 | 1.59 | 1.40 | 0.76 | 0.65 | 0.51 | 1.03 |
| 43 | 1.27 | 1.53 | 0.69 | 0.58 | 0.57 | 0.63 |
| 44 | 1.40 | 1.46 | 0.86 | 0.78 | 0.63 | 0.72 |
| 45 | 1.47 | 1.63 | 0.83 | 0.45 | 0.55 | 0.83 |
| 46 | 1.46 | 1.19 | 0.63 | 0.49 | 0.58 | 0.69 |
| 47 | 1.84 | 1.42 | 0.62 | 0.62 | 0.49 | 0.75 |
| 48 | 1.81 | 1.91 | 0.78 | 0.51 | 0.65 | 0.96 |
| 49 | 1.66 | 1.49 | 0.58 | 0.63 | 0.60 | 0.86 |
| 50 | 1.92 | 1.78 | 0.76 | 0.82 | 0.62 | 1.17 |
| 51 | 2.07 | 1.57 | 0.80 | 0.68 | 0.66 | 1.01 |
| 52 | 1.62 | 1.77 | 0.86 | 0.61 | 0.48 | 0.80 |
